# Determinants of Severe Orthodontic Treatment Need Using Clinically Categorized Occlusal Features

**DOI:** 10.3390/dj14060342

**Published:** 2026-06-04

**Authors:** Anwar S. Alhazmi

**Affiliations:** Department of Preventive Dental Sciences, College of Dentistry, Jazan University, Jazan 828172, Saudi Arabia; aalhazmi3@jazanu.edu.sa

**Keywords:** orthodontic treatment need, dental aesthetic index, screening, logistic regression, malocclusion, IOTN

## Abstract

**Background/Objectives:** Systematic assessment of orthodontic treatment needs is essential for prioritizing care in public health programs. The Dental Aesthetic Index (DAI) provides a composite severity score; however, understanding which individual occlusal traits drive severe treatment needs has a practical screening value. This study aimed to determine which occlusal features, categorized using established clinical thresholds, independently associated with severe orthodontic treatment need. **Methods:** Pretreatment records of 292 patients (141 men, 151 women; aged 13–42 years) were analyzed. The outcome was severe treatment need (DAI ≥ 31). Ten DAI components were categorized using IOTN-DHC severity cutpoints; Angle and skeletal classifications were also assessed. Candidate predictors (*p* < 0.20) were entered into multivariate logistic regression with backward elimination. Discrimination was assessed by ROC analysis; internal validation used bootstrap resampling and cross-validation. **Results:** Of the 292 patients, 141 (48.3%) had severe treatment needs. Six occlusal features were independently associated with severe treatment need (all *p* < 0.001): overjet severity (adjusted odds ratio [OR] = 412.4; 95% confidence interval [CI]: 82.1–2072.1), missing anterior teeth (OR = 29.3; 95% CI: 8.6–99.5), open bite severity (OR = 13.4; 95% CI: 4.6–39.5), diastema (OR = 7.4; 95% CI: 2.4–22.5), molar cusp relationship (OR = 4.7; 95% CI: 2.3–9.5), and anterior spacing (OR = 2.9; 95% CI: 1.5–5.4). The area under the ROC curve was 0.971 (optimism-corrected: 0.968), and 90.8% of the patients were correctly classified by the final model (sensitivity, 89.4%; specificity, 92.1%). Angle classification, skeletal classification, sex, age, and crowding were not included in the final model. **Conclusions:** Six clinically categorized occlusal features assessed by visual inspection were independently associated with severe orthodontic treatment need in this explanatory analysis, with overjet severity as the dominant determinant. Categorical severity grades based on established clinical thresholds retained strong discriminative information (optimism-corrected AUC = 0.968), suggesting that simplified assessments may be sufficient for the initial screening. Dental and skeletal classifications did not provide an independent predictive value beyond that of occlusal features. External validation is needed before clinical implementation.

## 1. Introduction

Malocclusion ranks among the most common oral conditions globally, with clinically significant forms affecting more than half of most populations studied [1]. In Saudi Arabia, reported prevalence rates range widely—from 47% to 93%—depending on the index and the region [2,3,4]. Beyond the clinical dimension, malocclusion carries a psychosocial burden: individuals who receive orthodontic treatment consistently report better oral health–related quality of life than those who do not [5,6].

When demand for orthodontic care outstrips supply—as it does in many public health settings—some form of systematic triage is needed [7]. The Dental Aesthetic Index (DAI), adopted by the WHO, combines clinical measurements with aesthetic weighting into a single composite score that reflects malocclusion severity and treatment urgency [8,9,10]. The Index of Orthodontic Treatment Need–Dental Health Component (IOTN-DHC) takes a different approach, classifying need on the basis of the single worst occlusal feature [11]. Both indices are well validated, yet both impose practical burdens: the DAI requires precise millimeter measurements and a weighted regression formula, while the IOTN-DHC demands memorization of a complex grading hierarchy [12].

Previous studies using logistic regression have identified DAI components that predict treatment needs. De Oliveira Meira et al. [13] found that DAI severity levels were significantly associated with adolescents’ aesthetic concerns. Silva et al. [14] reported that diastema, irregularities, mandibular overjet, open bite, and molar relationships influence the determination of treatment needs in a non-white population. De Melo et al. [15] showed that crowding and spacing are the anterior occlusal conditions most strongly associated with aesthetic concerns in adolescents. Carranza et al. [16] identified missing teeth (odds ratio [OR] = 8.9) and maxillary irregularities (OR = 8.6) as the strongest risk factors for orthodontic treatment. However, these studies used raw DAI component categories as predictors. No study has tested whether categorization based on established IOTN-DHC severity thresholds, which are designed to reflect clinical treatment urgency, can identify the key determinants of severe treatment needs.

Previous studies from this institution have described the distribution of malocclusion severity and its association with Angle’s classification in a Saudi population [17]. However, the independent determinants of severe treatment needs have not been examined using multivariate modeling. This study aimed to determine which occlusal features, categorized using established IOTN-DHC severity thresholds, are independently associated with severe orthodontic treatment needs and whether dental and skeletal classifications add predictive value beyond occlusal traits alone.

## 2. Materials and Methods

This retrospective analytical cross-sectional study used pretreatment orthodontic records from the Department of Orthodontics, College of Dentistry, Jazan University, Saudi Arabia. This study was approved by the Standing Committee for Scientific Research Ethics of Jazan University (reference number: REC-41/1-015, date of approval: 18 November 2021). The study was reported following the guidelines of Transparent Reporting of a Multivariable Prediction Model for Individual Prognosis or Diagnosis (TRIPOD) (see Supplementary Materials: File S1) [18,19].

Records of 292 patients (141 males and 151 females, aged 13–42 years) with complete permanent dentition referred for orthodontic evaluation were included. All pretreatment records meeting the inclusion criteria were included, representing a consecutive series of eligible patients. No a priori sample size calculation was performed; instead, sample adequacy for logistic regression was confirmed post hoc using the events-per-variable (EPV) criterion—141 events across six final predictors yielded an EPV of 23.5, well above the recommended minimum of 10 [20]. Post hoc power analysis (G*Power 3.1) confirmed that with 292 subjects and a baseline event probability of 0.483, the study had >99% power to detect an odds ratio of 2.0 at the α = 0.05 level. The inclusion criteria were complete pretreatment records (study casts, orthopantomographs, and lateral cephalometric radiographs), permanent dentition, and no history of prior orthodontic treatment.

### Examiner Calibration and Reliability

Three examiners independently measured all DAI components from pretreatment study casts following the WHO Oral Health Survey methods [8]. Intra-examiner reliability was assessed by re-measurement of 10 randomly selected casts after a two-week interval, and inter-examiner reliability was assessed across all three examiners. The intraclass correlation coefficients (ICC) for continuous variables [21] ranged from 0.969 to 0.995 (intra-examiner) and 0.964 to 0.990 (inter-examiner), indicating excellent agreement. Cohen’s and Fleiss’ kappa values for categorical variables [22] ranged from 0.908 to 1.000 (intra-examiner) and 0.902 to 1.000 (inter-examiner), indicating almost perfect agreement (all *p* < 0.001).

DAI scores were calculated from pretreatment study casts using the standard World Health Organization (WHO) regression equation, which incorporates 10 occlusal traits: “(visible missing teeth × 6) + (crowding) + (space) + (diastema × 3) + (anterior maxillary misalignment) + (anterior mandibular misalignment) + (anterior maxillary overjet × 4) + (anterior mandibular overjet × 4) + (anterior vertical open bite × 4) + (anteroposterior molar relationship × 3) + 13” [8,9]. Angle classification (Class I, Class II division 1, Class II division 2, and Class III) was determined from the study casts. Skeletal classification (Classes I, II, and III) was determined using lateral cephalometric radiographs based on the ANB angle.

The binary outcome was severe orthodontic treatment need, defined as DAI ≥ 31, combining the “treatment highly desirable” (DAI 31–35) and “treatment mandatory” (DAI ≥ 36) categories [10]. The interpretation and classification based on DAI scores are as follows: score ≤ 25, little or no treatment need; 26–30, treatment elective; 31–35, treatment highly desirable; and ≥36, treatment mandatory [8,9,10].

Continuous DAI components were categorized into clinical severity grades using established IOTN-DHC cutpoints [11] as follows: overjet, none (≤3 mm), mild (4–5 mm), and severe (≥6 mm); open bite, none (0 mm), mild (1–2 mm), and severe (≥3 mm); missing anterior teeth, none, one, two, or more. Ordinal predictors were coded as integer scores reflecting increasing severity (0, 1, and 2). Anterior crowding and spacing were coded as none, one segment, or two segments, and diastema as absent or present, following DAI conventions. The molar cusp relationship was coded as normal or no occlusion (0), half-cusp deviation (1), and full-cusp deviation (2), following the DAI anteroposterior molar relationship component [9]. Angle classification was entered as an indicator variable with Class I as the reference category. Skeletal classification, age, and sex were assessed as potential predictors. This yielded 14 predictor terms representing 10 clinical variables that were entered into univariate screening. Because the binary outcome (DAI ≥ 31) is derived from the same occlusal components entered as predictors, this analysis should be understood as an explanatory model identifying the relative contribution of each component to overall severity, rather than a transportable predictive model.

Descriptive statistics were calculated for all variables and stratified by treatment need group. Chi-square tests were used to evaluate associations between categorical variables and treatment need, and age was compared using the Mann–Whitney U test. Each candidate predictor was then entered into a univariate logistic regression to estimate crude odds ratios (ORs) with 95% confidence intervals (CIs). Variables reaching *p* < 0.20 were carried forward into a multivariate logistic regression model, in line with the approach recommended by Hosmer and Lemeshow [23]. Ordinal predictors with three severity levels (0, 1, 2) were entered as linear terms; with only three ordered categories reflecting a clinical severity gradient, the linear-in-the-logit assumption is parsimonious and consistent with the underlying dose–response relationship. Backward elimination was applied, removing variables with *p* > 0.05 at each step until only statistically significant predictors remained. The events-per-variable ratio was monitored throughout to ensure model stability, with a minimum of ten events per predictor considered adequate [20].

The model’s ability to discriminate between patients with and without severe treatment need was quantified using the area under the receiver operating characteristic curve (AUC) [24]. Calibration was assessed with the Hosmer–Lemeshow goodness-of-fit test [23]. Sensitivity, specificity, positive predictive value (PPV), negative predictive value (NPV), and overall accuracy were calculated at a probability cut-off of 0.50. To gauge how well the model would perform on new data, internal validation was carried out using 1000 bootstrap resamples (to estimate the optimism-corrected AUC) and 10-fold stratified cross-validation (to assess stability across data subsets) [25]. Backward elimination was chosen over penalized variable selection methods (e.g., LASSO) to preserve the interpretability of individual odds ratios, which is important for clinical translation. Because quasi-complete separation was observed for the overjet severity variable, Firth’s penalized logistic regression was run as a sensitivity analysis to check whether the odds ratio estimates held up under regularization [26]. All analyses were performed in Python 3.11 (StatsModels 0.14, scikit-learn 1.3), with statistical significance set at *p* < 0.05.

## 3. Results

Of the 292 patients, 141 (48.3%) had severe treatment needs (DAI score ≥ 31). The mean age was 23.6 ± 6.0 years (Table 1). No significant differences were found between the treatment groups in terms of sex (*p* = 0.55) or skeletal classification (*p* = 0.56). Age showed a marginal trend (*p* = 0.065) but was not retained in the multivariate analysis. The distribution of molar cusp relationships differed significantly between the groups, with full-cusp deviation present in 48.9% of patients with severe treatment needs compared to 27.2% of those without (*p* < 0.001). Overjet severity showed the strongest univariate association with treatment need; 98.0% of patients without excess overjet had no severe treatment need, whereas 100% of patients with severe overjet (≥6 mm) required treatment.

Table 2 presents the results of the univariate logistic regression. Nine of the 14 candidate predictor terms met the *p* < 0.20 screening criterion: overjet severity (crude OR = 58.8; *p* < 0.001), molar cusp relationship (OR = 2.3; *p* < 0.001), missing anterior teeth (OR = 5.9; *p* < 0.001), diastema (OR = 2.5; *p* = 0.001), open bite severity (OR = 2.4; *p* = 0.002), anterior spacing (OR = 1.9; *p* < 0.001), Angle Class II division 1 (OR = 6.3; *p* < 0.001), Angle Class II division 2 (OR = 0.3; *p* = 0.11), and age (OR = 0.96; *p* = 0.065). Sex, crowding, and Angle Class III, skeletal Class II, and skeletal Class III did not meet the screening threshold (all *p* > 0.20).

After backward elimination of the nine screened variables, age (*p* = 0.72), Angle Class II division 2 (*p* = 0.62), and Angle Class II division 1 (*p* = 0.26) were sequentially removed. The final model retained six predictors, all of which were occlusal components of the DAI (all *p* < 0.001; Table 3). Overjet severity was the dominant determinant (adjusted OR = 412.4; 95% CI: 82.1–2072.1), followed by missing anterior teeth (OR = 29.3; 95% CI: 8.6–99.5), open bite severity (OR = 13.4; 95% CI: 4.6–39.5), diastema (OR = 7.4; 95% CI: 2.4–22.5), molar cusp relationship (OR = 4.7; 95% CI: 2.3–9.5), and anterior spacing (OR = 2.9; 95% CI: 1.5–5.4). Angle classification did not independently contribute when these six occlusal features were included in the model. The events-per-variable ratio was 141/6 = 23.5, which exceeded the recommended minimum of 10 [20].

**Figure 1:**
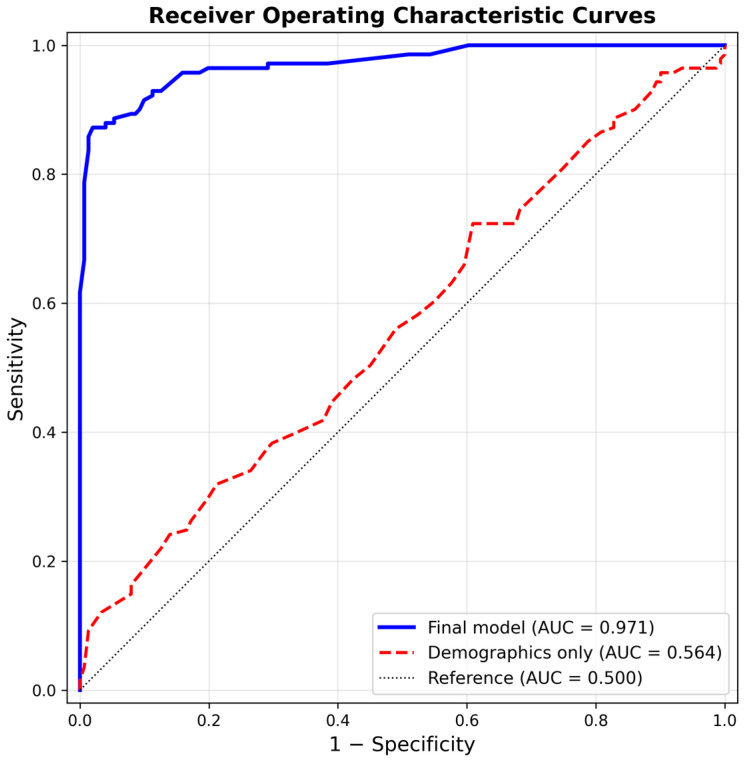
Receiver operating characteristic curves comparing the final six-variable model (AUC = 0.971) with the demographics-only model (AUC = 0.564). The diagonal line represents chance discrimination (AUC = 0.50). AUC indicates area under the curve.

The final model achieved an AUC of 0.971 compared to 0.564 for demographics alone (Figure 1). The Hosmer–Lemeshow test indicated adequate calibration (χ^2^ = 7.99; df = 8; *p* = 0.435). At a probability cut-off of 0.50, the model correctly classified 90.8% of the patients (sensitivity, 89.4%; specificity, 92.1%; PPV, 91.3%; NPV, 90.3%).

Bootstrap internal validation (1000 resamples) yielded a mean optimism of 0.004 (0.4% of the apparent AUC), resulting in an optimism-corrected AUC of 0.968. Ten-fold cross-validation produced a mean AUC of 0.972 ± 0.024 (range: 0.921–1.000), confirming minimal overfitting. Firth’s penalized logistic regression, conducted to address quasi-complete separation in the overjet variable, produced attenuated but directionally consistent estimates: overjet severity (Firth OR = 275.5; 95% CI: 62.3–1218.3), missing anterior teeth (OR = 22.2), open bite severity (OR = 11.3), diastema (OR = 6.5), molar cusp relationship (OR = 4.1), and anterior spacing (OR = 2.7). All predictors remained significant, and the AUC remained unchanged at 0.971, confirming that the findings were stable across estimation methods.

## 4. Discussion

The final model identified six occlusal features that, together, discriminated between patients with and without severe treatment need with an AUC of 0.971 and an overall classification accuracy above 90%. Overjet severity emerged as the single strongest driver, followed by missing anterior teeth, open bite, diastema, molar cusp relationship, and anterior spacing. All six are DAI components; none of the non-DAI variables—Angle classification, skeletal class, crowding, age, or sex—added independent predictive value once these occlusal features were in the model.

Overjet severity was the strongest determinant by a wide margin, ranking first in both the standard and penalized analyses. The wide confidence interval around the standard estimate (OR = 412.4; 95% CI: 82.1–2072.1) reflects quasi-complete separation—every patient with severe overjet (≥6 mm) fell into the severe treatment need group—and the point estimate should be interpreted as indicating the direction and relative dominance of this variable rather than a precise effect size. Firth’s penalized regression produced a substantially attenuated estimate (OR = 275.5; 95% CI: 62.3–1218.3) that remained the largest in the model, confirming overjet severity as the leading determinant regardless of estimation method. Given the quasi-complete separation, the magnitude of both estimates should be regarded as reflecting the strength of the association in ordinal terms rather than as a clinically calibrated effect size. De Oliveira Meira et al. [13] similarly reported that DAI-assessed overjet was the occlusal trait most strongly tied to aesthetic impact in a population-based sample of 1172 adolescents. By contrast, De Melo et al. [15] found that crowding and spacing mattered more for aesthetic concerns, whereas in our clinic-based sample overjet dominated the normative treatment need assessment. This discrepancy points to a clinically relevant distinction: overjet is the main driver of normative need, but anterior alignment weighs more heavily in patients’ own aesthetic perceptions [15,27]. Clinicians should keep in mind that what the index flags as most severe may not be what the patient considers most bothersome [28].

Missing anterior teeth ranked second among the determinants. Carranza et al. [16] reported that missing teeth were the strongest risk factor (odds ratio [OR] = 8.9) for treatment needs in adolescents. In the IOTN-DHC hierarchy, missing teeth requiring pre-restorative orthodontics represent grades 4–5 severity [11], and the DAI formula assigns the highest weight (×6) to this component [9], reinforcing the importance of this variable across indices. Open-bite severity ranked third, matching the findings of Silva et al. [14], who found that open bites were significantly associated with severe malocclusion in a non-white population. These results also align with the established psychosocial impact of these occlusal traits on oral health-related quality of life [5,29].

The molar cusp relationship also proved to be an independent predictor (OR = 4.7). This makes clinical sense: the DAI anteroposterior molar component captures the degree of cusp discrepancy—normal, half-cusp, or full-cusp—which is essentially the same sagittal dimension that the Angle classification describes. Once the molar cusp variable was in the model, Angle Class II division 1 dropped out (*p* = 0.26), indicating that these two variables were tapping into overlapping information and the simpler ordinal measure was sufficient. The practical takeaway is straightforward: a chairside check of molar cusp deviation using the DAI convention provides enough information for screening, and the formal Angle classification adds nothing beyond what the molar cusp variable already captures. Along similar lines, skeletal classification from cephalometric radiography did not improve prediction, suggesting that a lateral cephalogram is not necessary just for identifying patients with severe treatment need.

Crowding was not a significant predictor (*p* = 0.86); however, it was the most common trait in this population (82%). Crowding was nearly ubiquitous in this orthodontic referral population, indicating that it did not discriminate between the severity groups. The DAI formula assigns no multiplication weight to the crowding component [9], and our findings support this choice. These results differ from those of studies conducted in the general population, in which crowding frequency varies more substantially between those with and without treatment needs [2,30].

What distinguishes this analysis is the use of IOTN-DHC severity thresholds to categorize continuous measurements rather than arbitrary data-driven cutoff points. This approach bridges the two major orthodontic indices and produces clinically interpretable categories. The DAI is a more globally acceptable index as proposed in the WHO oral health basic survey methods [8]. However, DAI-based treatment indications should serve only as a guide because traits not captured by the index, such as skeletal discrepancies and impacted teeth, also influence orthodontic treatment decisions [17]. Recent work using item response theory has similarly demonstrated the correlation between dental health and aesthetic components of malocclusion indices [31]. A clinician does not need to measure overjet to the nearest 0.5 mm; determining whether overjet is absent, mildly increased, or severely increased is sufficient for screening purposes.

In terms of discrimination, the model (AUC = 0.971; optimism-corrected 0.968) performs well relative to other prediction models in orthodontics [32,33]. Bootstrap validation showed negligible overfitting (optimism = 0.004), and 10-fold cross-validation produced stable results across folds (mean AUC = 0.972 ± 0.024). However, the AUC should be interpreted as an upper bound of discriminative ability inherent to the index structure, not as a transportable measure of predictive accuracy, given the mathematical link between the predictors and the DAI-derived outcome. Even so, the model offers a practical advantage: it pinpoints which specific occlusal features carry the most weight in the composite score, thereby simplifying the screening task. The finding also suggests that the IOTN-DHC severity thresholds retain enough discriminative information to be useful when applied to DAI component data.

Two findings remain clinically informative regardless of the circularity limitation discussed above. First, only six of the ten DAI components reached independent significance, revealing which traits most strongly push a patient from moderate into severe treatment need. Second, recoding continuous measurements into IOTN-DHC severity grades did not appreciably reduce discrimination, which suggests that simplified categorical assessments may be workable in screening contexts. Confirming this will require external validation against an independent outcome measure such as the IOTN-DHC.

From a service-planning perspective, these results are timely. Saudi Arabia’s National Transformation Program under Vision 2030 is expanding dental coverage into underserved areas where specialist orthodontic assessment may not be readily available. A screening tool based on the six features identified here—overjet severity, missing anterior teeth, open bite, diastema, spacing, and molar cusp relationship—could be applied by primary care dentists or even in school-based programs, since it relies on categorical severity grades that require visual inspection rather than precise instrumentation.

**Figure 2:**
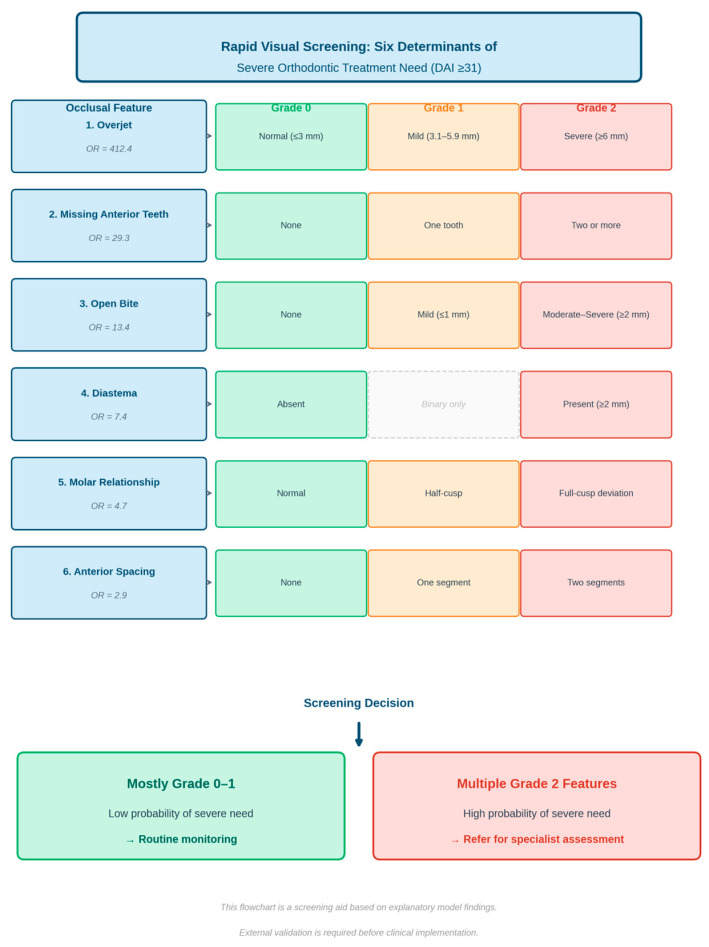
Rapid visual screening flowchart based on the six independent determinants of severe orthodontic treatment need. Each occlusal feature is graded into severity categories (Grade 0/1/2) derived from IOTN-DHC thresholds, allowing chairside classification by visual inspection without precise measurements. Green boxes indicate normal findings (Grade 0), orange boxes indicate mild severity (Grade 1), and red boxes indicate severe findings (Grade 2). Patients presenting with multiple Grade 2 features should be referred for specialist orthodontic assessment.

A key practical advantage of the present approach is that it replaces the precise millimeter measurements and weighted regression formula required by the traditional DAI with visual categorical grades derived from IOTN-DHC thresholds. The six determinants identified here—overjet severity, missing anterior teeth, open bite, diastema, molar cusp relationship, and anterior spacing—can each be assessed by visual inspection and basic clinical judgment in a matter of seconds. A screening flowchart based on these six features (Figure 2) could enable a single examiner to classify treatment urgency in under one minute per patient, making large-scale school-based and community screening programs substantially more time-efficient than programs relying on full DAI or IOTN-DHC scoring. This reduction in administrative and clinical workload is particularly relevant for non-specialist dental staff who may lack training in index-based assessment.

Several limitations should be acknowledged. The mathematical dependency between the DAI-derived outcome and the DAI component predictors represents the principal methodological constraint of this study; the reported AUC reflects the internal coherence of the index rather than the model’s ability to predict an independent clinical outcome. The retrospective, single-center design and the fact that all patients were drawn from an orthodontic referral clinic further constrain generalizability. Because all patients were referred for orthodontic evaluation, the prevalence of severe malocclusion in this sample (48.3%) is likely higher than in the general Saudi population, where population-based studies report figures of 24–37% [2,3,4,32,34]; the model’s positive predictive value would be expected to decrease in lower-prevalence settings. The small numbers of patients with Angle Class II Division 2 (*n* = 12) and Class III (*n* = 13) left limited power to test these classifications as independent predictors. Although internal validation pointed to minimal overfitting, external validation in an independent cohort—ideally using an independent outcome criterion such as the IOTN-DHC—is essential before these findings can inform clinical screening decisions. Until such validation is completed, the results should be regarded as hypothesis-generating rather than ready for direct clinical implementation. Future work should also examine whether occlusal features not captured by the DAI, such as posterior crossbite and deep overbite, add predictive value [17,35].

## 5. Conclusions

Six occlusal features—overjet severity, missing anterior teeth, open bite severity, diastema, molar cusp relationship, and anterior spacing—were independently associated with severe orthodontic treatment need when categorized using IOTN-DHC clinical thresholds. Overjet severity was by far the strongest single determinant. These categorical severity grades retained strong discriminative information (optimism-corrected AUC = 0.968) within the explanatory framework of this study, suggesting that simplified visual assessments may be sufficient for initial screening. However, because the outcome was derived from the same index as the predictors, external validation against an independent outcome criterion is needed to establish transportable predictive accuracy. Neither dental nor skeletal classification added independent value once the six occlusal features were accounted for. These results support further investigation of DAI components recoded with IOTN-DHC severity grades as a potential screening framework. However, direct clinical implementation should be deferred until the model has been validated externally against an independent outcome measure in a population-based sample. If validated, this categorical approach could serve as a practical benchmark for community dentistry programs, enabling non-specialist staff to triage orthodontic referrals efficiently, thereby optimizing resource allocation and reducing the clinical workload associated with full index-based assessment.

## Figures and Tables

**Table 1:** Sample characteristics of 292 patients stratified by treatment need (DAI < 31 vs. ≥31).

Variable	Total (*N* = 292)	No Need (*n* = 151)	Severe Need (*n* = 141)	*p* Value
Age (years), mean ± SD	23.6 ± 6.0	24.2 ± 5.7	22.9 ± 6.2	0.065 †
**Sex,** ***n*** **(%)**				**0.55**
Male	141 (48.3)	76 (50.3)	65 (46.1)	
Female	151 (51.7)	75 (49.7)	76 (53.9)	
**Overjet severity,** ***n*** **(%)**				<0.001 *
None (≤3 mm)	204 (69.9)	148 (98.0)	56 (39.7)	
Mild (4–5 mm)	69 (23.6)	3 (2.0)	66 (46.8)	
Severe (≥6 mm)	19 (6.5)	0 (0.0)	19 (13.5)	
**Missing anterior teeth,** ***n*** **(%)**				<0.001 *
None	224 (76.7)	140 (92.7)	84 (59.6)	
One	35 (12.0)	11 (7.3)	24 (17.0)	
Two or more	33 (11.3)	0 (0.0)	33 (23.4)	
**Anterior crowding,** ***n*** **(%)**				**0.57**
None	53 (18.2)	25 (16.6)	28 (19.9)	
One segment	80 (27.4)	45 (29.8)	35 (24.8)	
Two segments	159 (54.5)	81 (53.6)	78 (55.3)	
**Anterior spacing,** ***n*** **(%)**				<0.001 *
None	127 (43.5)	84 (55.6)	43 (30.5)	
One segment	98 (33.6)	42 (27.8)	56 (39.7)	
Two segments	67 (22.9)	25 (16.6)	42 (29.8)	
**Diastema,** ***n*** **(%)**				0.001 *
Absent	225 (77.1)	127 (84.1)	98 (69.5)	
Present	67 (22.9)	24 (15.9)	43 (30.5)	
**Open bite severity,** ***n*** **(%)**				<0.001 *
None (0 mm)	230 (78.8)	131 (86.8)	99 (70.2)	
Mild (1–2 mm)	37 (12.7)	14 (9.3)	23 (16.3)	
Severe (≥3 mm)	25 (8.6)	6 (4.0)	19 (13.5)	
**Molar cusp relationship,** ***n*** **(%)**				<0.001 *
Normal	47 (16.1)	30 (19.9)	17 (12.1)	
Half-cusp	107 (36.6)	66 (43.7)	41 (29.1)	
Full-cusp	110 (37.7)	41 (27.2)	69 (48.9)	
No occlusion	28 (9.6)	14 (9.3)	14 (9.9)	
**Angle classification,** ***n*** **(%)**				<0.001 *
Class I	70 (24.0)	51 (33.8)	19 (13.5)	
Class II division 1	197 (67.5)	76 (50.3)	121 (85.8)	
Class II division 2	12 (4.1)	11 (7.3)	1 (0.7)	
Class III	13 (4.5)	13 (8.6)	0 (0.0)	
**Skeletal classification,** ***n*** **(%)**				**0.56**
Skeletal I	93 (31.8)	44 (29.1)	49 (34.8)	
Skeletal II	109 (37.3)	60 (39.7)	49 (34.8)	
Skeletal III	90 (30.8)	47 (31.1)	43 (30.5)	
DAI score, mean ± SD	31.3 ± 9.3	24.3 ± 3.7	38.8 ± 7.6	<0.001 †

* Chi-square test; † Mann–Whitney U test.

**Table 2:** Univariate logistic regression: crude odds ratios for candidate predictors of severe orthodontic treatment need (DAI ≥ 31).

Predictor	Crude OR	95% CI	*p* Value	Selected ‡
Age (years)	0.96	0.93–1.00	0.065	Yes
Sex (female vs. male)	1.18	0.75–1.88	0.470	No
Overjet severity (ordinal)	58.76	17.87–193.20	<0.001	Yes
Missing anterior teeth (ordinal)	5.90	3.23–10.75	<0.001	Yes
Anterior crowding (ordinal)	0.97	0.72–1.31	0.857	No
Anterior spacing (ordinal)	1.89	1.39–2.57	<0.001	Yes
Diastema (present vs. absent)	2.45	1.42–4.20	0.001	Yes
Open bite severity (ordinal)	2.56	1.42–4.62	0.002	Yes
Molar cusp relationship (ordinal)	2.32	1.69–3.19	<0.001	Yes
Angle Class II division 1	6.34	3.55–11.31	<0.001	Yes
Angle Class II division 2	0.34	0.09–1.29	0.114	Yes
Angle Class III	0.66	0.21–2.06	0.471	No
Skeletal Class II	0.81	0.50–1.30	0.385	No
Skeletal Class III	1.05	0.65–1.69	0.849	No

OR, odds ratio; CI, confidence interval. ‡ Variables with *p* < 0.20 were selected for multivariate analysis.

**Table 3:** Final multivariate logistic regression model: adjusted odds ratios for determinants of severe orthodontic treatment need (DAI ≥ 31), with Firth’s penalized estimates for comparison.

Predictor	Adjusted OR	95% CI	*p* Value	Firth OR §	Firth 95% CI
Overjet severity	412.4	82.1–2072.1	<0.001	275.5	62.3–1218.3
Missing anterior teeth	29.3	8.6–99.5	<0.001	22.2	7.4–66.8
Open bite severity	13.4	4.6–39.5	<0.001	11.3	4.0–31.5
Diastema (present)	7.4	2.4–22.5	<0.001	6.5	2.3–18.9
Molar cusp relationship	4.7	2.3–9.5	<0.001	4.1	2.1–8.1
Anterior spacing	2.9	1.5–5.4	<0.001	2.7	1.5–4.9

OR, odds ratio; CI, confidence interval. All predictors *p* < 0.001. § Firth’s penalized logistic regression conducted to address quasi-complete separation observed for overjet severity. Model fit: Hosmer–Lemeshow χ^2^ = 7.99, df = 8, *p* = 0.435; Nagelkerke R^2^ = 0.828; −2 Log Likelihood = 121.3. Events per variable = 23.5 (141 events/6 predictors).

## Data Availability

The data presented in this study are available upon reasonable request from the corresponding author owing to privacy restrictions related to patient records.

## Supplementary Materials

The following supporting information can be downloaded at: https://www.mdpi.com/article/10.3390/dj14060342/s1, File S1: TRIPOD Checklist for reporting a prediction model development study.
