# Supplementary material for: Determinants of Severe Orthodontic Treatment Need Using Clinically Categorized Occlusal Features
Source: Dent J (Basel). 2026 Jun 4;14(6):342. doi: 10.3390/dj14060342 (PMC13298014; doi:10.3390/dj14060342)
Supplement: Supplementary file 1 [file dentistry-14-00342-s001.zip › Additional_file_S1_TRIPOD_Checklist.pdf]

Additional file S1

TRIPOD Checklist for Prediction Model Development

Study: Determinants of Severe Orthodontic Treatment Need Using Clinically Categorized Occlusal Features

Author: Anwar Alhazmi

Type of study: Prediction model development

Checklist: *Collins GS, Reitsma JB, Altman DG, Moons KGM. Transparent Reporting of a multivariable prediction model for Individual Prognosis Or Diagnosis (TRIPOD): The TRIPOD Statement. Ann Intern Med. 2015;162(1):55–63.*

Item	Checklist Item	Page	Reported as
**Title and Abstract**			
1	Identify the study as developing a multivariable prediction model, the target population, and the outcome to be predicted.	1	Title: 'Determinants of Severe Orthodontic Treatment Need Using Clinically Categorized Occlusal Features'
2	Provide a summary of objectives, study design, setting, participants, sample size, predictors, outcome, statistical analysis, results, and conclusions.	1	Structured abstract with Background, Methods, Results, Conclusions
**Introduction**			
3a	Explain the medical context and rationale for developing the prediction model.	1–2	Background paragraphs 1–4: malocclusion prevalence, DAI/IOTN-DHC indices, prior logistic regression studies, gap identification
3b	Specify the objectives, including whether the study describes the development of the model.	2	'This study aimed to determine which occlusal features...are independently associated with severe orthodontic treatment needs'
**Methods**			
4a	Describe the study design or source of data.	2	'This retrospective analytical cross-sectional study used pretreatment orthodontic records...'
4b	Specify the key study dates.	2	Not explicitly dated; records from the Department of Orthodontics, Jazan University
5a	Specify key elements of the study setting.	2	Department of Orthodontics, College of Dentistry, Jazan University, Saudi Arabia
5b	Describe eligibility criteria for participants.	2–3	Inclusion: complete pretreatment records, permanent dentition, no previous orthodontic treatment
5c	Give details of treatments received, if relevant.	—	Not applicable (pretreatment records)
6a	Clearly define the outcome that is predicted by the prediction model.	3	Severe orthodontic treatment need defined as DAI ≥31
6b	Report any actions to blind assessment of the outcome to be predicted.	—	Not applicable; DAI score calculated from measured components
7a	Clearly define all predictors used in developing the model.	3	Ten DAI components categorized using IOTN-DHC cutpoints, Angle classification, skeletal classification, age, sex; 14 predictor terms from 10 clinical variables
7b	Report any actions to blind assessment of predictors for the outcome and other predictors.	2–3	Three examiners independently measured DAI components; reliability assessed
8	Explain how the study size was arrived at.	2	Census of eligible records; EPV criterion confirmed (141 events / 6 predictors = 23.5, exceeding minimum of 10)
9	Describe how missing data were handled.	—	Complete records required for inclusion; no missing data

10a	Describe how predictors were handled in the analyses.	3	Continuous variables categorized using IOTN-DHC thresholds; ordinal coding (0, 1, 2); Angle classification as indicator variables
10b	Specify type of model, all model-building procedures, and method for internal validation.	3–4	Multivariate logistic regression; univariate screening ($P < .20$); backward elimination; 1000 bootstrap resamples; 10-fold cross-validation; Firth's penalized regression for sensitivity analysis
10c	For validation, describe how the predictions were calculated.	—	Development only; internal validation via bootstrap and cross-validation
10d	Specify all measures used to assess model performance and, if relevant, to compare multiple models.	3–4	AUC, Hosmer–Lemeshow test, sensitivity, specificity, PPV, NPV, overall accuracy, optimism-corrected AUC
11	Provide details on how risk groups were created, if done.	3	Binary outcome: DAI ≥31 (severe/handicapping) vs DAI <31
**Results**			
12	Describe the flow of participants through the study.	4	292 patients included; 141 (48.3%) had severe treatment needs
13a	Describe the characteristics of the participants.	4	Table 1: characteristics stratified by treatment need group
13b	For validation, show a comparison with the development data of the distribution of important variables.	—	Development only
14a	Specify the number of participants and outcome events in each analysis.	4	292 patients; 141 events; EPV = 23.5
14b	If done, report the unadjusted association between each candidate predictor and outcome.	4	Table 2: univariate logistic regression with crude ORs for all 14 candidate predictor terms
15a	Present the full prediction model to allow predictions for individuals.	4–5	Table 3: final model with adjusted ORs, 95% CIs, and P-values for all six predictors; Firth's penalized estimates
15b	Explain how to use the prediction model.	6–7	Discussion: six-variable screening profile described; categorical severity grades require only visual inspection
16	Report performance measures (with CIs) for the prediction model.	5	AUC = 0.971; optimism-corrected AUC = 0.968; sensitivity 89.4%; specificity 92.1%; accuracy 90.8%; H-L $P = .435$
**Discussion**			
17	Discuss any limitations of the study.	7	Single-center retrospective design; clinic-based sample; small subgroups for Class II/2 and III; internal validation only; mathematical dependency between predictors and outcome
18	For validation, discuss the results with reference to performance in the development data, and any other validation data.	5, 7	Bootstrap optimism = 0.004; 10-fold CV AUC = 0.972 ± 0.024; Firth's penalized estimates confirmed robustness
19a	For validation, discuss the potential clinical use of the model and implications for future research.	7	Practical screening tool for primary care and school-based programs; external validation needed
19b	Discuss the implications for clinical use and future research.	7	Vision 2030 service planning; simplified categorical screening; external validation with independent outcome (IOTN-DHC) recommended
20	Provide information about the availability of supplementary resources.	7	Datasets available from corresponding author; TRIPOD checklist (this file)
**Other Information**			

21	Give the source of funding and the role of the funders for the present study.	7	'This research did not receive any specific grants from funding agencies'
22	Provide registration information.	—	Not registered (retrospective observational study)
